# The Time Has Come to Explore Plasma Biomarkers in Genetic Cardiomyopathies

**DOI:** 10.3390/ijms22062955

**Published:** 2021-03-14

**Authors:** Nienke M. Stege, Rudolf A. de Boer, Maarten P. van den Berg, Herman H. W. Silljé

**Affiliations:** Department of Cardiology, University Medical Center Groningen, University of Groningen, Hanzeplein 1, AB43, 9713 GZ Groningen, The Netherlands; n.m.stege@umcg.nl (N.M.S.); r.a.de.boer@umcg.nl (R.A.d.B.); m.p.van.den.berg@umcg.nl (M.P.v.d.B.)

**Keywords:** plasma biomarkers, genetic cardiomyopathy, early detection, noncoding RNA, cardiac autoantibodies, HCM, DCM, ACM

## Abstract

For patients with hypertrophic cardiomyopathy (HCM), dilated cardiomyopathy (DCM) or arrhythmogenic cardiomyopathy (ACM), screening for pathogenic variants has become standard clinical practice. Genetic cascade screening also allows the identification of relatives that carry the same mutation as the proband, but disease onset and severity in mutation carriers often remains uncertain. Early detection of disease onset may allow timely treatment before irreversible changes are present. Although plasma biomarkers may aid in the prediction of disease onset, monitoring relies predominantly on identifying early clinical symptoms, on imaging techniques like echocardiography (Echo) and cardiac magnetic resonance imaging (CMR), and on (ambulatory) electrocardiography (electrocardiograms (ECGs)). In contrast to most other cardiac diseases, which are explained by a combination of risk factors and comorbidities, genetic cardiomyopathies have a clear primary genetically defined cardiac background. Cardiomyopathy cohorts could therefore have excellent value in biomarker studies and in distinguishing biomarkers related to the primary cardiac disease from those related to extracardiac, secondary organ dysfunction. Despite this advantage, biomarker investigations in cardiomyopathies are still limited, most likely due to the limited number of carriers in the past. Here, we discuss not only the potential use of established plasma biomarkers, including natriuretic peptides and troponins, but also the use of novel biomarkers, such as cardiac autoantibodies in genetic cardiomyopathy, and discuss how we can gauge biomarker studies in cardiomyopathy cohorts for heart failure at large.

## 1. Introduction

**Figure 1:**
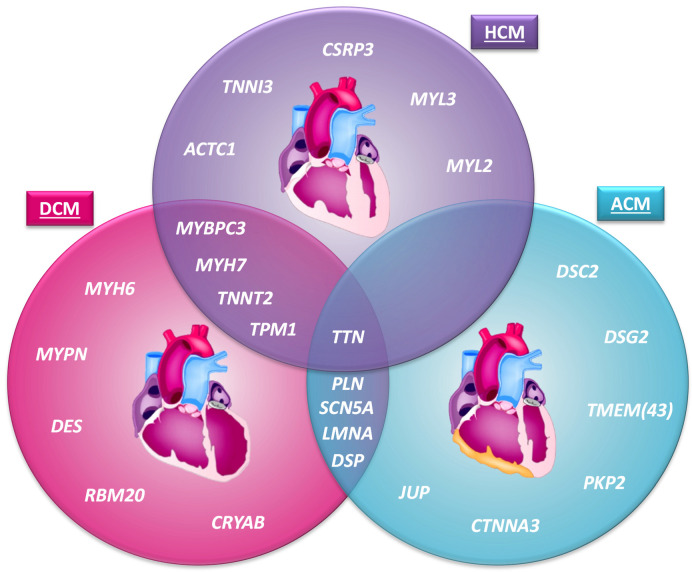
The most common genetic cardiomyopathies and a selection of the most frequently implicated genes. The hearts used in this figure are adapted from McCauley and Wehrens (2009) [23], licensed under a Creative Commons Attribution Non-Commercial Share Alike 3.0 Unported License (https://creativecommons.org/licenses/by-nc-sa/3.0/legalcode, accessed on 14 March 2021). ACM = arrhythmogenic cardiomyopathy; DCM = dilated cardiomyopathy; HCM = hypertrophic cardiomyopathy.

Genetic cardiomyopathy refers to a disease in which a pathogenic gene variant causes structural or functional abnormalities of the heart muscle, resulting in cardiac dysfunction. About 30 years ago, the first genetic cardiomyopathy gene mutation was described [1]. The identified pathogenic variant in *MYH7* (p.Arg403Glu) results in hypertrophic cardiomyopathy (HCM). This work pioneered a genetic era in which a large number of pathogenic cardiac gene variants have been identified as causing different forms of cardiomyopathies. Genetic cardiomyopathies are classified into groups: hypertrophic cardiomyopathy (HCM), dilated cardiomyopathy (DCM), arrhythmogenic cardiomyopathy (ACM), left ventricular non-compaction cardiomyopathy (LVNC) and restrictive cardiomyopathy (RCM) [2] (Figure 1), based on their cardiac phenotype observed by imaging or (post-mortem) histological findings.

It is important to stress that cardiomyopathies are not defined by a specific genetic mutation, but by specific morphological and functional cardiac alterations. HCM is characterized by left ventricular hypertrophy, unexplained by secondary causes, in the absence of left ventricular dilatation [3]. Although HCM is believed to be predominantly genetically determined, in a substantial proportion of patients the exact cause and/or pathogenic variant cannot be identified. For DCM this is even more complex and it is often regarded a mixed cardiomyopathy as it can have a genetic cause, but other factors contribute as well [2,4]. DCM is defined by the presence of ventricular enlargement and systolic dysfunction in the absence of left ventricular hypertrophy, and can have many causes [3]. It has been suggested that 20–50% of idiopathic DCM is a result of a genetic cause [5]. ACM is characterized by replacement of the ventricular myocardium with fibrofatty tissue and the presence of ventricular arrhythmias [3,6]. Although both ventricles can be affected, in many patients it is confined to the right ventricle, resulting in the sub classification of arrhythmogenic right ventricular cardiomyopathy (ARVC) [7]. In LVNC, a sponge-like left ventricular myocardium is present [8] and in RCM an abnormally rigid non-dilated left and/or right ventricle is present with severe diastolic dysfunction [3]. HCM and DCM are by far the most prevalent cardiomyopathies, with HCM having a prevalence of about 1/500 [9], and DCM between 1/2500–1/250 [10], although the exact prevalence of genetic DCM is uncertain. ACM has a prevalence of about 1/5000 [2,11], whereas the others (RCM, LVNC) can be classified as rare, and will not be discussed in this review [12,13].

The clinical classification, based on specific morphological and functional cardiac alterations, has existed since the time when the underlying (genetic) causes were still unknown. Imaging modalities, including echocardiography (Echo), cardiac magnetic resonance (CMR) and several other modalities [14], have therefore obtained a prominent role in the diagnosis and monitoring of patients [15,16,17,18,19,20]. Genetic testing has subsequently been included, but cardiac biomarkers have so far not received a prominent role in the diagnosis or prognosis of genetic cardiomyopathies. This in contrast to other cardiovascular diseases, including coronary artery disease (CAD) and heart failure (HF), in which cardiac troponins (cTns) and natriuretic peptides (NPs) have a prominent role in diagnosis [21]. Circulating biomarkers may provide information at both early and late stages of the disease process and could therefore be very useful for monitoring inherited disease [22]. The generally relatively small, single-center and observational studies in genetic cardiomyopathies have clearly hampered biomarker investigations in this field. With the increase in genetic (cascade) testing, cardiomyopathy cohorts have become larger and now provide an opportunity to perform biomarker studies. Although in this review we discuss biomarkers particularly in the context of genetic cardiomyopathies, in many of the described studies a distinction between genetic or other causes for the described cardiomyopathy was not apparent. This is a limitation for the proper interpretation of such studies. We anticipate that improved genetic screening will solve this caveat to a large degree in future cardiomyopathy investigations, and will improve study population definitions.

## 2. Genes Implicated in Cardiomyopathies

A large number of pathogenic gene variants have been identified and this number is likely to increase further. Although some genes can be linked to a specific cardiomyopathy, like HCM or ACM, other genes are associated with multiple forms of cardiomyopathy, often depending on the specific pathogenic variant, and on secondary disease triggers (Figure 1). For certain pathogenic variants, like the PLN Arg14del variant, disease may even develop in different directions, in this case being either ACM or DCM or both [24].

HCM is often caused by a single mutation in a sarcomere-associated gene [25] (Figure 1). Up to 50% of genetic HCM cases are explained by mutations in the *MYH7* or *MYBPC3* genes [26,27,28,29]. Other genes explain a far smaller proportion; for instance, mutations in *TNNT2*, *TNNI3* and *TPM1* account for less than 10% of the genetic HCM cases [26,27,30,31]. Even less frequently present, but established, gene mutations causal for HCM, are found in the genes *ACTC1*, *MYL2*, *MYL3*, *CSRP3* and Titin (*TTN*) [32,33,34,35]. All these pathogenic variants cause histological and morphological changes, in particular disarray and hypertrophy of cardiomyocytes, with interstitial fibrosis [36]. These changes are reflected by an impaired cardiac (diastolic) function, with typically normal to supranormal (65% to 70%) to elevated (>70%) left ventricular ejection fraction (LVEF), at least in the early stage of the disease, and a reduced end-systolic volume [36] due to extreme LV hypertrophy. 

The main pattern of inheritance in DCM is autosomal dominant and it has a high genetic heterogeneity [10,37,38]. Mutations are often found in genes encoding structural proteins such as sarcomeric, cytoskeleton and sarcolemma proteins (Figure 1). In 12–25% of the genetic DCM cases, mutations are present in *TTN*, encoding the largest sarcomeric protein, Titin [39]. Other DCM-related genes that also encode structural proteins are *TNNT2* (2–3%), *MYH7* (4–10%), *MYH6* (4%), *TPM1* (1–2%), *MYPN* (3–4%), *DES* (<1%) and *MYBPC3* [40,41,42]. DCM-causing mutations have also been found in the nuclear lamin A/C encoding gene *LMNA* (4–6%), in the *RBM20* gene (2–5%) encoding a nuclear RNA splicing factor, in ion-channel-related genes like *SCN5A* (2–3%) and *PLN*, in intercellular junction coding genes such as *DSP* (3–4%), and in the heat shock Alpha-crystallin B chain coding gene *CRYAB* [40,41,42,43]. Sarcomeric mutations may thus underlie both DCM and HCM. In DCM, the impaired processes often result in the loss of myofibril organization and cell death and as a consequence of dilatation of one or both ventricles, along with impaired contractility, defined by a left ventricular ejection fraction (LVEF lower than 45% or fractional shortening (FS) below 25% [18], with LV dilatation (classic DCM) or without it (non-dilating form of DCM).

ACM is also typically inherited in an autosomal dominant manner and is a genetically heterogeneous disorder. Many established disease-causing genes encode desmosomal proteins (Figure 1). The most common desmosomal coding genes are *PKP2* (25–40%), *DSP* (2–12%), *DSG2* (5–10%), *DSC2* (2–7%) and *JUP* [44,45,46]. Less common ACM genes are nuclear envelope coding genes *TMEM43* and *LMNA*, ion-channel-related genes including *PLN* and *SCN5A*, and *CTNNA3*, which encodes for alpha-T-catenin functions in cell–cell adhesion via its interaction with plakophilins [44,45,46]. The mutations in the desmosomal coding genes cause abnormalities in the desmosomes, resulting in the detachment of cardiomyocytes [2]. Therefore, the intracellular signal transduction is disturbed. Moreover, cardiomyocyte death and fibrofatty replacement are observed [2]. These pathological changes cause ventricular arrhythmias and ventricular dysfunction. 

## 3. Development of Cardiac Dysfunction

**Figure 2:**
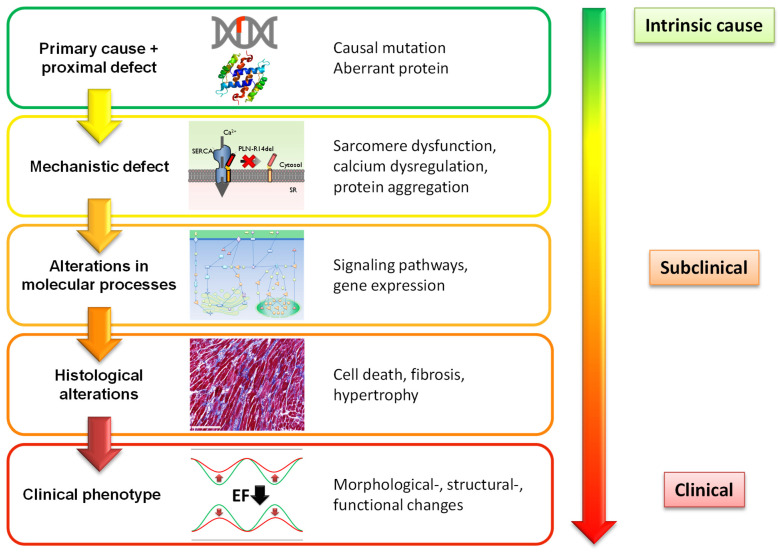
Suggested stages of disease development in genetic cardiomyopathy.

In genetic cardiomyopathies, the causal pathogenic variant and the direct proximal defect, such as a missing, truncated or misfolded protein, are often recognized. Moreover, based on the pathogenic variant, the expected clinical phenotype(s) can often be anticipated, even though the exact underlying molecular mechanisms and sequence of events remain in many cases a black box (Figure 2). In addition, the interaction with other environmental or (epi)genetic factors is incompletely understood [47]. These interactions may be important; for instance, for pathogenic desmosomal variants, a causal relation with exercise in the development of ACM has been described [48,49]. Due to these knowledge gaps, the time of disease onset and disease severity remain in most cases unpredictable [50].

**Figure 3:**
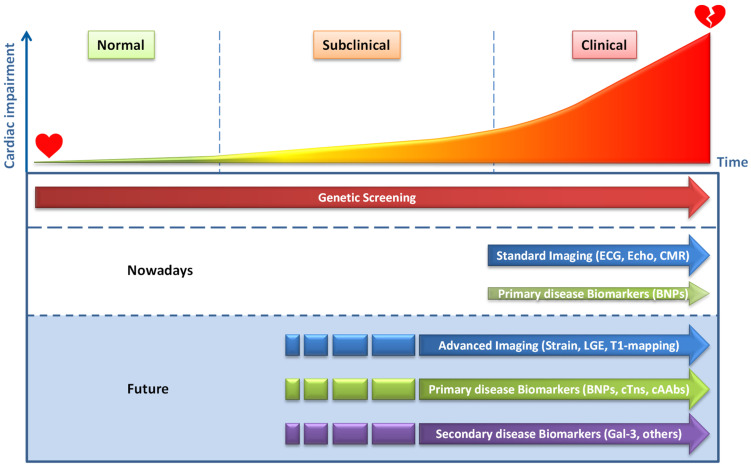
Moment of disease detection in relation to the level of cardiac impairment. ECG = electrocardiogram; Echo = echocardiography; BNPs = B-type natriuretic peptide and N-terminal pro BNP; LGE = late gadolinium enhancement, cTns= cardiac-specific Troponin I and T; cAAbs = cardiac autoantibodies; Gal-3 = galectin-3; Others include GDF15 and sST2.

However, we have obtained a framework that should allow us to improve our current knowledge and diagnostic performance. In line with previous suggestions [36], we propose a sequence of events in a mutation carrier that finally culminates in a clinical phenotype (Figure 2). Before cardiac structural and functional abnormalities become evident and can be detected, molecular and cellular changes will already have occurred in cardiomyocytes or other cardiac cells [50]. Calcium homeostasis, sarcomere function, metabolism and other cellular processes can be affected by pathogenic variants long before disease development becomes apparent (secondary mechanistic defects). These effects can be subtle and may not directly cause disease development, but can activate signaling pathways and trigger, among other processes, differential gene expression, like the fetal gene program [51]. Expression of atrial and B-type natriuretic peptides (ANP and BNP) are well-known examples of this fetal gene program and important cardiac-specific plasma biomarkers for HF. Ultimately, the activation of these pathways drives pathogenic processes, including cardiomyocyte hypertrophy, cell death (necrosis, apoptosis) and fibrosis (histological changes), and results in structural, morphological and functional alterations of the heart and final clinical presentation. Imaging techniques allow excellent detection of these later defects. CMR, together with late gadolinium enhancement (LGE) or T1 mapping can be used for the detection of cardiac fibrosis, and specific fibrotic patterns in cardiomyopathies have been described [52]. Advanced imaging techniques, like cardiac strain analyses, revealed that myocardial strain defects could already be detected in HCM and DCM mutation carriers before the development of left ventricular hypertrophy and overt contractile dysfunction on standard imaging [53,54,55]. These results show that abnormalities in myocardial mechanical properties precede the development of hypertrophy in HCM and support the multi-step model of disease development, as outlined in Figure 2. Based on this model of cardiomyopathy development, we suggest that besides advanced imaging, plasma biomarkers may allow the detection of early subclinical disease development and provide information on particular pathological processes in the heart (Figure 3). In this review, we will distinguish biomarkers that are predominantly derived from the heart (cardiac-specific biomarkers) and biomarkers that are also produced by other organs and tissues (non-cardiac-specific) [56]. As discussed, the former will primarily reflect cardiac disease, whereas the non-cardiac biomarkers will mostly reflect secondary disease development in cardiomyopathies. The various plasma biomarkers that will be discussed are also summarized in Table S1.

## 4. Cardiac-Specific Plasma Protein Biomarkers

Diagnosis and management of genetic cardiomyopathies predominantly relies on genetic testing, clinical symptoms, (ambulatory) ECG measurements and cardiac imaging to detect functional, structural and morphological alterations [15,16,17,18,19,20] (Figure 3). Cardiac-specific plasma biomarkers currently have no decisive role in the diagnosis and management of genetic cardiomyopathies. Nevertheless, cardiac-specific B-type natriuretic peptides (BNP, and the inactive but more stable N-terminal domain of BNP, NT-proBNP), which are actively secreted by cardiomyocytes upon cardiac wall stress, do provide clinical utility. BNP and NT-proBNP are the gold-standard biomarkers for HF and are included in the HF guidelines of the European Society of Cardiology (ESC) and the American College of Cardiology/American Heart Association (ACC/AHA) [57,58,59,60]. Since BNP or NT-proBNP can add prognostic information to standard risk factors for predicting sudden cardiac death (SCD) or sudden cardiac arrest (SCA), they are also included in guidelines for patients with structural heart disease and ventricular arrhythmias [17,20]. Moreover, elevated NT-proBNP and BNP levels in patients with structural heart disease are associated with the risk of ventricular tachyarrhythmias and are predictive for sudden cardiac death and ventricular arrhythmias [61,62]. A recent bioinformatics study revealed that BMP-10 appears to be the only actively secreted protein that shows a similar cardiac restrictive expression to that of NPs [63]. This protein clearly deserves attention, but except for a study showing that circulating BMP10 can identify patients at risk of recurrent atrial fibrillation after ablation [64], the biomarker potential of this protein has not been explored.

Cardiac-specific Troponin isoforms I and T (cTnI and cTnT), which are released upon cardiomyocyte cell death, are included in the definition for myocardial infarction and are indicated as plasma biomarkers in the acute coronary syndrome guidelines [65,66,67]. The development of highly sensitive cardiac troponin (hs-cTn) tests has provided a more precise definition of what is ‘normal or healthy’ (the 99th percentile, upper reference limit (URL)) and allows for the detection of low levels of cardiomyocyte cell death and provides utility beyond the detection of myocardial infarction [68]. Other cardiac-specific biomarkers that may identify cardiomyocyte cell death include heart-type fatty acid-binding protein (hFABP) and cardiac myosin-binding protein C3 (MyBPC3, also known as cMyC) [69,70]. It was recently shown that the detectability of cMyBPC3 in control plasma samples was superior to that of hs-cTnT, suggesting that MyBPC3 might be a better biomarker for detecting dormant cardiac disease, and this deserves future attention [71].

### 4.1. NPs and Troponins in HCM, DCM and ACM

Elevated levels of NPs and hs-cTn are associated with cardiovascular events, heart failure and death in HCM, and for prognostic purposes, laboratory tests for these biomarkers are recommended in the ESC HCM cardiomyopathy guidelines [15]. It has been shown that HCM patients have elevated plasma levels of NT-proBNP, BNP and cTnI in contrast to subclinical HCM mutation carriers [72,73]. This suggests an association of these biomarkers with hypertrophy development. Exercise strongly increased plasma BNP and cTn levels in HCM patients, suggesting that the combination with exercise could have a stronger discriminatory effect [73,74]. Interestingly, it was shown that exercise increased BNP plasma levels particularly in HCM patients with silent ischemia [75]. Importantly, BNP and cTn levels were also associated with fibrosis in HCM patients and cTn levels were predictive for the detection of extensive myocardial fibrosis in non-high risk patients with HCM [76,77]. Troponin measurements could therefore be used as a pretest to select patients for CMR imaging with LGE or T1 mapping to establish cardiac fibrosis.

Since DCM is classified as non-ischemic cardiac disease with cardiac dilatation and EF < 45%, the routine use of BNP and/or NT-proBNP will automatically apply to this HF condition. In ACM, NT-proBNP has been associated with RV dilatation and dysfunction [78,79]. Since HF development is the common final syndrome for most cardiomyopathy patients, routine BNP and NT-proBNP measurements will apply to those cardiomyopathy patients that develop HF symptoms [57,58,59,60].

In general, in cardiomyopathy patients with structural heart disease and/or clinical symptoms, elevated plasma levels of NT-proBNP, BNP and cTns are associated with a higher risk of cardiovascular events, HF and death [75,80,81,82,83,84].

### 4.2. BNPs and Troponins in Subclinical Disease 

It has been suggested that small elevations of NT-proBNP and BNP, far below the traditional cut-off points for HF, may be indicative of elevated cardiac stress and may be an early warning sign for people without any known cardiac disease [85]. Indeed, in community-based studies it has been shown that plasma NP levels predict the risk of death and cardiovascular events after adjustment for traditional risk factors, and excess risk was also apparent in natriuretic peptide levels that were below thresholds used to diagnose HF [86,87,88,89,90]. This indicates that small differences in NPs can already indicate disease development before clinical manifestation. Furthermore, cardiac troponins may have predictive value in the absence of known cardiac disease [90,91,92]. In the Multi-Ethnic Study of Atherosclerosis (MESA), using a cohort that was initially free of overt cardiovascular disease (CVD), hs-cTnT levels were shown to be associated with replacement fibrosis and progressive changes in left ventricular function [93]. It was therefore suggested that minor elevations of hs-cTnT may represent a biochemical signature of early subclinical cardiac disease, which may precede HF symptoms by years. Moreover, in a large population of healthy women (121,700 participants), baseline levels of NT-proBNP were associated with subsequent risk of SCD [94]. In addition, in a community-based population of elderly people, NT-proBNP provided information regarding the risk of SCD, beyond other traditional risk factors [95]. Interestingly, these data in the general population also appear to apply in the setting of inherited cardiomyopathies. Indeed, in a recent DCM study with LMNA cardiomyopathy, an elevated hsTnT level (>14 ng/L) was present in one third of the relatives of LMNA cardiomyopathy probands, before the onset of clinical symptoms [96]. This suggests that hsTnT could be an early marker and elevated levels should raise a red flag in such carriers. Elevated hsTnT plasma concentration was the earliest marker of carrier status in LMNA-related cardiomyopathy. Moreover, this study showed a strong, independent association between NT-proBNP levels and the occurrence of malignant ventricular arrhythmia among *LMNA* mutation carriers. The use of hsTn and NT-proBNP in subclinical DCM is supported by animal studies. DCM is one of the most common cardiac diseases in dogs [97]. In Doberman Pinschers, DCM is an inherited and slowly progressive disease, and biomarkers (NT-proBNP and hsTn) are widely accepted in the diagnosis of occult DCM in Doberman Pinschers [98]. A longitudinal study in Dobermans showed that plasma concentrations of NT-proBNP were increased in both dogs with DCM and in apparently healthy dogs (based on Echo and Holter monitoring) that developed DCM within 1.5 years after plasma sampling [99]. A similar kind of study revealed that elevated cTnI was also associated with DCM development (within 1.5 years’ follow-up) [100].

Altogether, BNPs and cTns clearly seem to have value in detecting subclinical cardiac disease (Figure 3), but longitudinal cohort studies in different patient groups are urgently needed to confirm this.

## 5. Non-Cardiac-Specific Plasma Protein Biomarkers

A number of non-cardiac-specific protein-based biomarkers have attracted a lot of attention in the field of cardiovascular disease during the last decade. In particular, proteins that play a role in modulating inflammatory and/or fibrotic responses, including galectin-3 (Gal-3), growth differentiation factor 15 (GDF15) and soluble suppression of tumorigenesis-2 (sST2). A number of excellent reviews describe the function and properties of these proteins [101,102,103,104,105,106,107]. Although their usefulness in clinical practice still needs to be confirmed, Gal-3 and sST2 have been included as biomarkers for myocardial fibrosis in the 2013 American College of Cardiology Foundation/American Heart Association (ACC/AHA) HF guidelines, for risk stratification as well as for prognosis in patients with moderate and severe HF (class IIb) [59].

Whether non-cardiac-specific biomarkers of fibrosis and inflammation can be used for detection of cardiac-specific fibrosis and inflammation remains uncertain [108]. These biomarkers were shown to be elevated in many other syndromes, like obesity, cancers, nephropathy and other diseases and in many cases plasma levels do not mirror cardiac production. Moreover, in animal studies, a direct correlation between cardiac function and fibrosis and Gal-3 and GDF15 plasma levels was lacking [56]. This may also partly explain the rather confusing results in cardiomyopathies as described below.

### 5.1. Non-Cardiac-Specific Plasma Biomarkers in HCM, DCM and ACM

Gal-3 plasma concentrations have been shown to be elevated in patients with HCM, which was also related to disease severity [109,110]. In an HCM cohort with mostly New York Heart Association (NYHA) class I patients, Gal-3 plasma levels were not elevated compared to controls [73]. This strongly suggests that in HCM, Gal-3 is predominantly related to HF severity, which is corroborated by the observation that no association between Gal-3 levels and LV hypertrophy exists in HCM patients with predominantly mild HF symptoms [73]. In addition, Gal-3 plasma levels did not correlate with LGE-detected fibrosis in HCM patients [73,77,111]. For GDF15, plasma levels were also associated with disease severity in HCM patients [112]. However, similarly to Gal-3, GDF15 was not different between HCM patients with or without LGE-detected fibrosis [77]. sST2 plasma levels have been shown to be elevated in HCM patients as compared to controls and were also associated with NYHA class [109,113]. One study showed that sST2 levels were not elevated in HCM patients, which most likely reflects the low NYHA classification of these patients [73]. One biomarker that appeared to mark myocardial fibrosis, in both subclinical HCM mutation carriers and HCM patients, was the propeptide of type I procollagen (PICP), but this was not confirmed in a later multicenter cohort study [72,73]. Some confounder effects were suggested for these different outcomes and these will require further investigations.

In DCM patients, plasma Gal-3 levels were elevated and also associated with cardiac fibrosis and were predictive for prognosis [111,114]. GDF-15 was shown to be associated with an increased risk of arrhythmic death in a small prospective study with 52 DCM patients [115]. Therefore, it was suggested that GDF-15 could provide additional information on top of LVEF, in identifying patients at risk of arrhythmic death. In end-stage DCM patients, GDF-15 plasma levels were strongly elevated compared to controls and were also correlated with myocardial fibrosis and kidney function [116]. Most interestingly, a strong decline in circulating GDF-15 was observed in these patients within 1 month of mechanical unloading (LVAD). However, the authors did not detect substantial cardiac GDF-15 mRNA and protein, suggesting that the heart was not an important source for elevated circulating GDF-15 in these patients. This is suggestive of extracardiac production of GDF-15 in heart disease, which is in line with recent animal studies [56]. Regarding sST2, the data are limited, but the available data revealed that it was not predictive for arrhythmic death in DCM, but was associated with all-cause mortality [115,117].

Circulating Gal-3 concentrations were significantly elevated in a study in ACM patients [118]. Moreover, the levels were higher in patients with ventricular tachycardia or ventricular fibrillation (VT/VF) than those without VT/VF and were predictive for ventricular arrhythmias in ACM patients with implantable defibrillators [118]. GDF15 and sST2 were shown to be elevated in ACM patients with biventricular involvement and the combined use of NT-proBNP, sST2 and GDF-15 showed the best prediction of LV involvement [119]. sST2 was associated with RV global strain and with left ventricular function in a study with 42 genotype positive ACM patients [120]. In this study, plasma levels of sST2 were higher in patients with ventricular arrhythmias than in patients without ventricular arrhythmias [120].

### 5.2. Non-Cardiac-Specific Plasma Biomarkers in Subclinical Heart Disease

There is currently no evidence that Gal-3, GDF15 and sST2 may have value in the detection of subclinical disease in genetic cardiomyopathies. Gal-3 and sST2 were not elevated in subclinical HCM mutation carriers and not even in HCM patients [73]. These non-specific biomarkers were only elevated in cardiomyopathies when severe clinical symptoms were already present. We have recently described that such biomarkers are much more abundantly expressed in other tissues, like adipose tissue, and their increased levels more likely reflect stress in other organs and tissues, either as a result of heart failure or due to the presence of other co-morbidities [56,108].

It is interesting to note that, although NT-proBNP and troponin were shown to be predictive for incident HF in the general population, the predictive value of sST2, Gal3, and other non-cardiac-specific markers is limited and some sex-specific differences have been observed [90,121]. Interestingly, longitudinal changes in Gal-3 concentrations appear to have stronger predictive value for future cardiovascular diseases as compared to single measurements [122,123]. Thus, sequential biomarker analysis may provide better resolutions in subclinical populations. Non-cardiac-specific biomarkers may therefore have some value, but we should bear in mind that these changes often reflect non-cardiac stress. (Figure 3). Multi-panel biomarker investigations might provide more direction in relation to the exact stressor. Of interest, a recent plasma proteomics study, combined with machine learning, revealed a panel of six non-cardiac-specific plasma peptides to be associated with HCM [124]. Moreover, five of these peptides (ALDOA-peptide, C3-peptide, GSTO1-peptide, RSU1-peptide and THBS1-peptide) showed a significant elevation in subclinical HCM carriers. Whether these elevated levels also have predictive power has not been investigated, and the sample size of subclinical patients (*n* = 16) was rather low.

## 6. Noncoding RNA Biomarkers

In addition to protein plasma biomarkers, circulating noncoding RNAs (ncRNAs) have attracted great interest during the last decade as potential new biomarkers in cardiovascular diseases [125,126,127,128]. Based on their length and shape, ncRNAs can be divided into three classes, microRNAs (miRNAs), circular RNAs (circRNAs) and long noncoding RNAs (lncRNAs) [129]. Both active secretion of ncRNAs into the circulation and passive leakage due to cell death have been reported. Active secretion also includes the secretion via exosomes, for which the heart is also a source [130]. However, we will not differentiate between exosome and non-exosome ncRNAs in the discussion below because it is poorly described in studies, the biomarker potential of exosome ncRNAs is still elusive [131] and the plasma contribution over free circulating ncRNAs is still vague [132,133,134,135,136].

Within heart disease, circulating ncRNAs have been studied mainly in relation to myocardial infarction (MI), which predominantly involves the passive leakage of cardiac- and muscle-specific ncRNAs [71,128,137]. The cardiac-restricted expression of miR-208 and miR-499 has made them excellent candidates for the detection of cardiac injury [126,137]. In comparison with cTns, it was shown that miRNA levels rose faster than troponin, but also normalized much faster and failed to identify patients with MI that initially presented with low troponin values [71,137]. Moreover, in HF no changes in plasma levels were observed, suggesting that these miRNAs are excellent for detecting acute cardiac damage, but their rapid clearance makes them less suitable for detecting low-grade chronic cardiomyocyte cell death, as may occur in (sub)clinical stages in cardiomyopathies [138].

### 6.1. ncRNA Biomarkers in HCM, DCM and ACM

Although circulating levels of cardiac-specific miR-208 and miR-499 were not associated with HCM [139], several other miRNAs (i.e., miR-199a-5p, -27a and -29a) did show a correlation with hypertrophy parameters in the HCM group. Moreover, one of these, miR-29a, was significantly associated with both hypertrophy and fibrosis. Another study showed that miRNAs, which individually only had moderate diagnostic value for diffuse myocardial fibrosis in HCM, provided a good diagnostic value when used in combination [140]. Interestingly, that study also presented miR-29a as a diagnostic fibrosis biomarker, and it has been shown to act as a regulator of cardiac fibrosis in mouse studies [141]. Whether elevated blood plasma concentrations of miR-29a in HCM are a result of active secretion by the heart or mainly due to passive leakage from other tissues requires further investigation [142]. Two circRNAs, circDNAJC6 and circTMEM56, were shown to be negatively correlated with echocardiographic parameters for obstruction in the left ventricular outflow tract (hypertrophic obstructive cardiomyopathy, HOCM) and therefore may serve as markers for disease severity in these patients [143].

Unfortunately, data on circulating miRNA studies in DCM are very limited. A study in pediatric DCM patients did show a difference in miRNA expression levels (miR-155, miR-639, miR-636 and miR-646) between children with recovered ventricular function and children who did not recover [144], but since recovery is uncommon in adult DCM patients, it is difficult to translate these findings to the adult population. In a study with adult DCM patients, plasma miR-423-5p levels were shown to be positively correlated with the levels of NT-proBNP [145], but like in HCM, more studies are urgently needed.

Recently, six differentially expressed plasma miRNAs (miR-122-5p, miR-133a-3p, miR-133b, miR-142-3p, miR-182-5p, and miR-183-5p) were identified in ACM patients [146]. Three miRNAs were also differentially expressed in other cardiomyopathies [146], suggesting that these are not disease-specific. Interestingly, miR-133a-3p, miR-133b and miR-142-3p were also differentially expressed in non-affected family members of the ACM probands, which may indicate predictive power in subclinical mutation carriers, but this will require a prospective study.

### 6.2. ncRNA Biomarkers in Subclinical Heart Disease 

Whether ncRNAs may have potential in detecting subclinical disease in mutation carriers will require prospective follow-up studies. At this stage, the miRNA biomarkers for cardiomyocyte cell death (miR-208 and miR-499) appear to be less promising than troponins or MyBPC3 for detecting low levels of sustained cell death and replacement fibrosis. The low plasma levels of these cardiac-specific miRNA biomarkers also appear to hamper detection [71]. Similarly, circRNAs showed low plasma levels despite having a high abundance in cardiac tissue, and no changes after myocardial injury were observed [71]. Protein injury plasma biomarkers therefore still appear to be superior. Nevertheless, some miRNAs may be used for the early detection of diffuse fibrosis, in particular, miR-29a, which controls cardiac fibrosis and shows differential plasma levels in HCM. Although single ncRNAs do not seem to have predictive power in subclinical cardiac disease, ncRNA multi-panel biomarkers may have this power [146,147,148].

## 7. Autoantibodies

Antibodies against self-antigens, so-called autoantibodies (AAbs), are another potential source of plasma biomarkers. In inflammatory diseases, including myocarditis, AAbs against self-derived epitopes can often be detected [149]. The functional relevance of these AAbs is still unclear and unraveling the contributions of AAbs to disease development is difficult due to the complex nature of inflammatory diseases. Not only in myocarditis, but also in peripartum cardiomyopathy, for instance, a cardiac disease believed to involve a strong genetic component, elevated levels of anti-cardiac Troponin I (anti-cTnI) and anti-cardiac sarcomeric myosin (anti-MHC) were measured in 46% of the patients [150].

### 7.1. Autoantibody Biomarkers in HCM, DCM and ACM

Some small HCM cohort studies have been performed in the past, revealing, among other effects, an increase in the number of patients with elevated AAbs against G-protein-coupled β1 and Muscarin-2 receptors (β1-AAb and M2-AAb) [151,152,153]. A recent study with 134 HCM patients (and 40 controls) confirmed these past results and also showed that the concentration of M2-AAb in HCM patients with a family history of SCD or atrial fibrillation was significantly higher [154]. In addition, antibodies against the molecular chaperone calreticulin (CRT) have been described in HCM and in DCM [155]. This antibody is also associated with systemic lupus [156,157], but the relation with cardiac disease in this setting has not been investigated.

In DCM patients, anti-heart autoantibodies (AHAs), which are autoantibodies directed against heart tissue, have been described [158,159]. Not only have these AHAs been detected, in a study in which 592 asymptomatic first- or second-degree relatives of 169 DCM probands were investigated, it was even shown that AHAs were independent predictors of disease development within five years’ follow-up [160]. This finding led to the inclusion of AHAs in the diagnostic criteria for DCM relatives, in a position statement of the ESC working group on myocardial and pericardial diseases [4]. A major drawback for the screening of AHAs in patient plasma samples is, however, the requirement of cardiac or muscle tissue slides from healthy human donors and concomitant microscopic analysis. Therefore, rather than detecting antibodies targeting whole heart tissue, the detection of either a specific autoantibody against a cardiac restrictive protein or the detection of a selection of antibodies that provide a specific cardiac autoantibody (cAAb) signature will provide better applicability. It will therefore be pivotal to identify the epitopes to which these antibodies are generated and convert this into a routine screening platform for cAAb profile detection, like an enzyme-linked immunosorbent assay (ELISA). This adheres to the criteria for organ and disease-specificity, as mentioned by the position statement of the ESC working group on myocardial and pericardial diseases [4]. In line with this principle, specific-cAAbs against cTnI have been detected in the plasma of DCM patients [161] and a promising cAAb profile has been suggested for the detection of Brugada syndrome (BrS) [162], as discussed below. This indicates that specific cAAbs or cAAb profiles may have promising clinical biomarker potential.

Several recent studies also indicate the potential of AAbs in the diagnosis of ACM. Chatterjee et al. evaluated the biomarker potential of antibodies to cardiac desmosomal cadherin proteins in ACM and stated that AAbs to a specific desomosomal protein, desmoglein-2 (DSG2), are a sensitive and specific biomarker for ACM [163]. The anti-DSG2 antibodies were present in the sera of all of the ACM patients (a total of 37), whereas they were absent in 31 and faintly present in 1 out of 32 control sera [163]. As discussed by others, it still has to be proven whether this marker is ACM-specific, and measurements in sera from patients with other cardiac diseases were lacking [164]. In another interesting study, serum from ACM patients was tested for the presence of AHAs and for anti-intercalated disk autoantibodies (AIDAs) by immunofluorescence microscopy using human atrium and skeletal muscle tissue. This revealed a higher frequency of AHAs and AIDAs in probands (37 individuals) and clinically affected relatives (42 individuals), compared to sera from patients with non-inflammatory cardiac disease and ischemic heart failure and sera from healthy blood donors [165]. An association with family history and with features of disease severity was also reported [165]. This further confirms the presence of cardiac disease-specific AAbs, but as discussed above, it will be important to identify the specific antigenic determinants (epitopes) in order to allow the routine detection of disease-specific cAAb profiles. In this regard, it is highly interesting that Chatterjee et al. recently identified a cAAb profile for Brugada syndrome (BrS), and evaluated its diagnostic potential [162]. The cAAb biomarker profile involved antibodies against four proteins and turned out to be highly sensitive and specific for BrS patients [162]. The antibodies against cardiac and skeletal α-actins keratin-24 and connexin-43 were consistently present in the sera of patients with BrS and were absent in healthy controls and in patients with hypertrophic, dilated and arrhythmogenic cardiomyopathies [162]. Although independent confirmation is still needed, this finding underscores the value of investigating the biomarker potential of cAAb profiles for diagnosing a specific type of cardiomyopathy [166]. A role for cAAbs specific to one of the arrhythmia syndromes is therefore also projected in a recent expert consensus statement [167]. The four proteins to which the AAbs were directed turned out to be abnormally expressed in BrS. The targeted proteins were aggregated in BrS, in contrast to the diffuse reticular or speckled patterns observed in healthy myocardium [162]. Abnormally folded proteins or protein aggregate formation, caused by genetic variants, may therefore provoke an auto-immune response in which AAbs are produced. This might enable a directed search for disease-specific cAAb profiles based on the information of abnormally expressed proteins in specific genetic cardiomyopathies, for instance, desmin-positive and CRYAB-R102G-positive protein aggregates in desminopathy [168,169], PLN-positive protein aggregates in PLN p.Arg14del cardiomyopathy [170] and TMEM43-positive protein aggregates in p.S358L TMEM43 ARVC [171].

### 7.2. Autoantibody Biomarkers in Subclinical Heart Disease

It is too early to compose general statements about the potential of AAbs in detecting subclinical disease in mutation carriers, and additional studies will be required. The observation that AHAs can predict disease development in asymptomatic relatives of DCM probands is promising and has been acknowledged by the suggested inclusion of AHAs in diagnostic criteria for DCM relatives [4,160]. However, for broad clinical applications, disease-specific epitopes have to be identified in order to enable diagnostic screening for specific cAAbs or cAAb profiles using common detection platforms. A role for biomarkers to discriminate between specific arrhythmia syndromes is also anticipated [167]. Moreover, in ACM, the data on AAbs are rapidly expanding and it is not unlikely that such antibodies will also allow for the detection of subclinical disease. Since protein biomarker studies showed that cardiac specificity is of importance for biomarker potential, we believe that a cardiac-specific cAAb or a cAAb profile will have favorable qualities over single non-cardiac-specific AAbs. We therefore suggest specific cAAbs or cAAb profiles as potential early biomarkers for detecting disease onset in pathogenic variant carriers (Figure 3). Further investigations in HCM, DCM and ACM are required in order to confirm this.

## 8. Future Directions and Challenges

**Figure 4:**
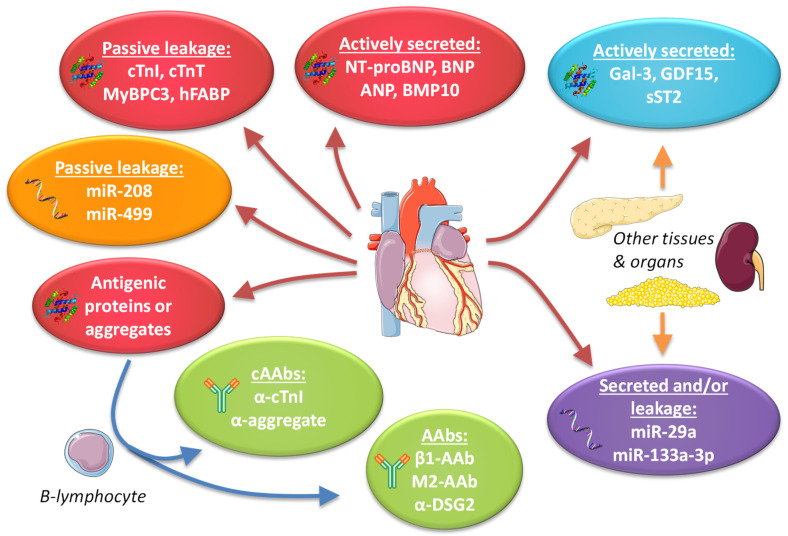
Overview of the current spectrum of potential biomarkers in genetic cardiomyopathies, including examples of biomarker proteins, noncoding RNAs (ncRNAs) and antibodies. Red/orange ovals indicate proteins/ncRNAs from the heart. Blue/purple: ovals indicate proteins/ncRNAs from multiple tissues. Green ovals indicate antibodies. AAbs = autoantibodies, cAAbs = cardiac autoantibodies, BNP = B-type natriuretic peptide, ANP = atrial natriuretic peptide, BMP10 = bone morphogenetic protein 10, Gal-3 = galectin-3, GDF15 = growth differentiation factor 15, sST2 = soluble suppression of tumorigenesis-2, β1 = G-protein coupled β1 receptor, M2 = muscarin-2 receptor, DSG2 = desmoglein-2, cTnI = cardiac troponin I, cTnT = cardiac troponin T, MyBPC3 = cardiac myosin-binding protein C3, hFABP = heart-type fatty acid-binding protein. Components of the figure are derived from Servier Medical Art (https://smart.servier.com/, accessed on 14 March 2021), licensed under a Creative Commons Attribution 3.0 Unported License (https://creativecommons.org/licenses/by/3.0/, accessed on 14 March 2021).

The current spectrum of potential biomarkers consists predominantly of passively leaked or actively secreted proteins, ncRNAs or antibodies generated against proteins released from the heart (Figure 4). In the latter case, the detection of autoantibodies targeting a cardiac-restricted protein or detection of a selective set of autoantibodies that provides a disease specific cardiac autoantibody (cAAb) signature would be most valuable. Based on the current data, plasma protein biomarkers and cAAbs have further advanced as biomarkers for cardiac disease as compared to ncRNAs. The ncRNA biomarker field is, however, relatively young and may still produce some surprises. At this stage, the detection of the low levels of plasma ncRNAs seems to be a major hurdle in this field and improvements in detection methods are needed. Moreover, although some plasma ncRNAs do have high cardiac specificity, this appears to be limited to ncRNAs that are released upon cell death (miR-208 and miR-499). To our knowledge, ncRNAs that are selectively and specifically secreted by cardiomyocytes, similarly to proteins like BNP, have not yet been identified. Such ncRNAs, if they exist, could be a major asset. At this stage, it appears that single ncRNAs have little predictive power and panels of ncRNAs are mainly being explored. In combination with protein plasma biomarkers, these ncRNAs may provide additional utility, as was recently shown for the identification of non-acute HF [172].

Although the cardiac-specific biomarkers BNP, NT-proBNP and cTns have proven their value in structural cardiac disease, their utility in subclinical disease still needs to be proven. Numerous studies in the general population have shown that elevated plasma levels have predictive value. This might be even more true for carriers of a genetic cardiomyopathy variant. In *LMNA* DCM mutation carriers, as well as in dog models for DCM, the potential of cTns and NPs to predict disease onset has been shown [96,99,100]. Similarly, anti-heart autoantibodies were independent predictors of disease development in genotype-positive relatives of DCM patients [160] and these antibodies have been recommended as a diagnostic test for these relatives [4]. Identification of specific cAAb profiles will, however, be necessary to convert this into generally applicable screening platforms. Furthermore, in relatives of ACM patients, the detection of cAAbs could have promising value in disease prediction. Finally, in non-high-risk HCM patients, cTns haven been shown to be indicative of myocardial fibrosis and can therefore be used to select patients for CMR with LGE or T1 mapping [77]. This indicates that cardiac-specific biomarkers could have value in subclinical monitoring and in the monitoring of non-high-risk patients, but defining the selection criteria (cut-off values) will be a major challenge. In this respect, changes in concentrations, rather than absolute value, might be considered, which would require the comparison of serial (annual) measurements instead of interpreting single measurements [173]. The use of these biomarkers (NPs, cTns) in the older population may be limited, since their levels are influenced by age, obesity and kidney function [174]. However, for relatively young mutation carriers this should be less of an issue. Correspondingly it has been shown that elevated cTn levels have much more predictive value in young subjects [93]. Together, this indicates that cardiac-specific biomarkers or antibodies can have value in disease prediction in mutation carriers and in non-high-risk cardiomyopathy patients. Longitudinal cohort studies will be needed to prove this.

In contrast to cardiac-specific biomarkers, non-cardiac-specific protein biomarkers Gal-3, GDF15 and sST2 appear to be elevated only if an overt clinical phenotype has developed in the different cardiomyopathies. This elevation most likely reflects a secondary effect as a result of diminished cardiac function and hemodynamics, causing stress in distal organs and tissues and subsequent extra-cardiac production of these biomarkers in these tissues. This explains why these biomarkers have strong prognostic value in cardiovascular disease. Moreover, common cardiovascular risk factors, like obesity and smoking, may affect the plasma levels of these biomarkers and hence their levels may also be an indication of other comorbidities. Since other underlying disease can also be a secondary driver in disease onset in cardiomyopathy, the levels of these non-cardiac-specific biomarkers should be carefully interpreted, as well as in subclinical disease. Thus, rather than being primary cardiac disease biomarkers, we prefer to designate them as secondary non-cardiac biomarkers.

**Figure 5:**
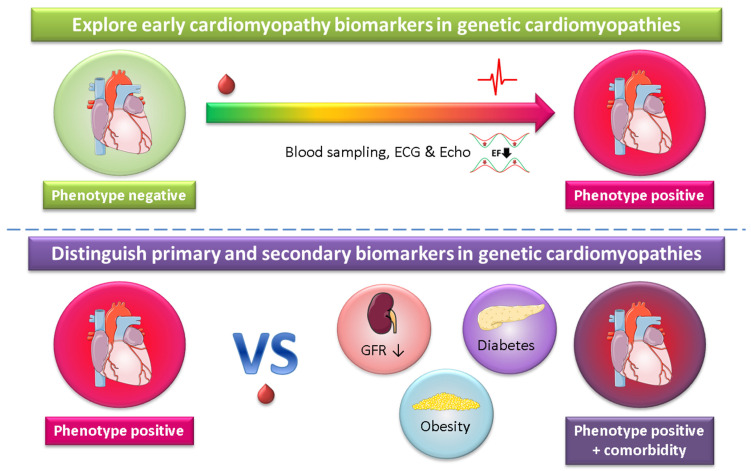
Genetic cardiomyopathy as a cardiac disease-specific model to explore biomarkers. ECG = electrocardiography, echo = echocardiography, EF = ejection fraction, GFR = glomerular filtration rate (a measure of kidney function). Components of the figure are derived from Servier Medical Art (https://smart.servier.com/, accessed on 14 March 2021), licensed under a Creative Commons Attribution 3.0 Unported License (https://creativecommons.org/licenses/by/3.0/, accessed on 14 March 2021).

Studies towards plasma biomarkers in genetic cardiomyopathy have been hampered for a long time because of the absence of large cohorts. However, due to genetic cascade screening, the number of identified carriers with specific pathogenic variations has strongly increased and the time has come to study this in larger cohorts. As an example, in 2012 a Dutch founder mutation (p.Arg14del) in the phospholamban (*PLN*) gene [24] was described, and 8 years later we already have more than 1000 registered carriers with a specific mutation. This will pave the way for biomarker studies in genetic cardiomyopathies. Not surprisingly, several prospective observational plasma biomarker studies have recently been described or registered at clinicaltrials.gov that include large cohorts (>250 subjects) with inherited cardiac conditions [175,176,177,178,179,180,181]. These studies are, among other purposes, aimed at exploring early cardiomyopathy biomarkers and will also provide information about primary and secondary biomarkers, as outlined in Figure 5. A major advantage of studying plasma biomarkers in genetic cardiomyopathy is the well-established primary cause of cardiac disease and the reduced disturbance of other cardiovascular risk factors and comorbidities, like diabetes and smoking. A high incidence of cardiac disease development is another advantage of investigating inherited disease carriers.

## 9. Conclusions

Genetic screening, together with standard imaging techniques, is the current clinical diagnostic practice for genetic cardiomyopathies. Cardiac-specific plasma protein biomarkers like NT-proBNP, cTns and specific cAAbs or cAAb profiles do hold promise to provide additional prognostic value and to identify disease onset. The latter would be extremely helpful to monitor non-affected carriers. So far, most genetic cardiomyopathy biomarker studies have been small observational studies and have had limited predictive power. Genetic cardiomyopathy cohorts have substantially grown in the last decade and now allow longitudinal cohort studies. Such studies will hopefully soon prove the suitability of many biomarkers in disease prediction and may allow us to distinguish between primary cardiac disease biomarkers and secondary non-cardiac biomarkers. Although no specific treatments are available for most genetic cardiomyopathies, the rapid development of disease-specific and gene-based therapies may soon change this. Selecting carriers and defining the optimal time for treatment will then be the next challenge and a proper set of biomarkers may be of great value in such decisions.

## Supplementary Materials

The following are available online at https://www.mdpi.com/1422-0067/22/6/2955/s1, Table S1: Overview of the potential plasma biomarkers for genetic cardiomyopathies.
