# Supplementary material for: The Time Has Come to Explore Plasma Biomarkers in Genetic Cardiomyopathies
Source: Int J Mol Sci. 2021 Mar 14;22(6):2955. doi: 10.3390/ijms22062955 (PMC7998409; doi:10.3390/ijms22062955)
Supplement: Supplementary file 1 [file ijms-22-02955-s001.pdf]

Table S1: Overview of the potential plasma biomarkers for genetic cardiomyopathies. HF = heart failure, HCM = hypertrophic cardiomyopathy, DCM = dilated cardiomyopathy, ACM = arrhythmogenic cardiomyopathy, NPs = natriuretic peptides (ANP, BNP, NT-proBNP), cTns = cardiac specific troponins (cTnI, cTnT), Gal-3 = Galectin-3, GDF-15 = Growth differentiation factor 15, sST2 = soluble suppression of tumorigenesis-2, β1 = G-protein coupled β1 receptor, M2 = Muscarin-2- receptor, DSG2 = desmoglein-2, RV = right ventricle, LV = left ventricle, LGE = Late Gadolinium Enhancement, VT/VF = Ventricular Tachycardia/ Ventricular Fibrillation, LVAD = left ventricular assist device, BrS = Brugada syndrome, SCD = sudden cardiac death.

Name	Biomarker type	Biological function	CM type	Evidence	References
NPs	Cardiac specific Plasma protein	Vasodilatation, Diuresis	HCM	Elevated plasma levels in patients in contrast to subclinical carriers. Levels are increased by exercise and are associated with fibrosis. In patients with structural heart disease and/or clinical symptoms elevated levels are associated with higher risk of cardiovascular events, HF and death.	[1–10]
			DCM	Already part of the HF (DCM) guidelines.	[11]
			ACM	Levels are associated with RV dilatation and dysfunction. In patients with structural heart disease and/or clinical symptoms elevated levels are associated with higher risk of cardiovascular events, HF and death.	[3,6–10,12,13]
cTns	Cardiac specific Plasma protein	Sarcomere function	HCM	Levels are elevated in patients, in contrast to the levels in subclinical carriers. Levels are predictive for myocardial fibrosis in non-high risk patients. In patients with structural heart disease and/or clinical symptoms elevated levels are associated with higher risk of cardiovascular events, HF and death.	[1–3,6–10]
			DCM	In patients with structural heart disease and/or clinical symptoms elevated levels are associated with higher risk of cardiovascular events, HF and death.	[3,6–10]
			ACM	In patients with structural heart disease and/or clinical symptoms elevated levels are associated with higher risk of cardiovascular events, HF and death.	[3,6–10]
Gal-3	Non-cardiac specific Plasma protein	Fibrosis, Inflammation	HCM	Levels are elevated in patients and are related to disease severity. No elevation and no association with LV hypertrophy in patients with mild symptoms. Also no correlation with LGE detected fibrosis.	[1,5,14–16]
			DCM	Levels are elevated in patients and are also associated with cardiac fibrosis. Levels were predictive for prognosis	[16,17]
			ACM	Elevated levels in patients and higher levels in the presence of VT/VF. Levels were predictive for ventricular arrhythmias in patients with implantable defibrillators.	[18]
GDF-15	Non-cardiac specific Plasma protein	Inflammation, Remodeling, Cell death and growth	HCM	Levels are associated with disease severity. There was no correlation with LGE detected fibrosis.	[5,19]
			DCM	Levels are associated with increased risk of arrhythmic death. Levels are strongly elevated in end-stage patients and correlate with myocardial fibrosis and kidney function. Strong level decline within one month after LVAD.	[20,21]

			ACM	Elevated levels in patients with biventricular involvement.	[22]
sST2	Non-cardiac specific Plasma protein	Inflammation	HCM	Elevated levels in patients and associated with NYHA class.	[14,23]
			DCM	Levels are not predictive for arrhythmic death. Levels are associated with all-cause mortality	[20,24]
			ACM	Levels are elevated in patients with biventricular involvement and are associated with RV global strain and LV function. Higher levels are detected if ventricular arrhythmias are present.	[22,25]
miR-208	Cardiac specific Noncoding RNA	Cardiac development, Stress response	HCM	Levels are not associated with HCM	[26]
			DCM & ACM	Studies will be needed	
miR-499	Cardiac specific Noncoding RNA	Cardiac development, Stress response	HCM	Levels are not associated with HCM	[26]
			DCM & ACM	Studies will be needed	
miR-29a	Non-cardiac specific Noncoding RNA	Proliferation, Apoptosis, Differentiation, Fibrosis	HCM	Levels are associated with both hypertrophy and fibrosis	[26]
			DCM & ACM	Studies will be needed	
miR-133a-3p	Non-cardiac specific Noncoding RNA	Proliferation, Differentiation	HCM	Not differentially expressed in one study, more studies will be needed	[27]
			DCM	Differentially expressed in one study, more studies will be needed	[27]
			ACM	Differentially expressed in patients and non-affected family members	[27]
α-cTnI	Cardiac specific cAAbs	Inflammation	DCM	Independent predictor of disease development within 5 years follow up. Included in diagnostic criteria for DCM relatives	[28–30]
			HCM & ACM	Studies will be needed	
α-aggregate	Cardiac specific cAAbs	Inflammation	ACM	AHAs against cardiac α-actins, keratin-24, and connexin-43 in detected in BrS, while absent in healthy and HCM, DCM and ACM controls. Additional studies are required .	[31,32]
			HCM&DCM	Studies will be needed	
β1-AAb	Non-cardiac specific AAbs	Inflammation	HCM	Elevated levels in patients	[33]
			DCM & ACM	Studies will be needed	
M2-Aab	Non-cardiac specific AAbs	Inflammation	HCM	Elevated levels in patients. Concentration is higher in patients with a family history of SCD or atrial fibrillation.	[34,35]
			DCM & ACM	Studies will be needed	
α-DSG2	Non-cardiac specific AAbs	Inflammation	ACM	Sensitivity and specificity shown by one study (in which cardiac disease control groups lacked). Additional studies will be needed.	[36]
			HCM & DCM	Studies will be needed	

Supplemental References

1. Ho, J.E.; Shi, L.; Day, S.M.; Colan, S.D.; Russell, M.W.; Towbin, J.A.; Sherrid, M. V.; Canter, C.E.; Jefferies, J.L.; Murphy, A.; et al. Biomarkers of cardiovascular stress and fibrosis in preclinical hypertrophic cardiomyopathy. *Open Hear.* **2017**, doi:10.1136/openhrt-2017-000615.
2. Ho, C.Y.; López, B.; Coelho-Filho, O.R.; Lakdawala, N.K.; Cirino, A.L.; Jarolim, P.; Kwong, R.; González, A.; Colan, S.D.; Seidman, J.G.; et al. Myocardial Fibrosis as an Early Manifestation of Hypertrophic Cardiomyopathy. *N. Engl. J. Med.* **2010**, doi:10.1056/nejmoa1002659.
3. Nakamura, T.; Sakamoto, K.; Yamano, T.; Kikkawa, M.; Zen, K.; Hikosaka, T.; Kubota, T.; Azuma, A.; Nishimura, T. Increased plasma brain natriuretic peptide level as a guide for silent myocardial ischemia in patients with non-obstructive hypertrophic cardiomyopathy. *J. Am. Coll. Cardiol.* **2002**, doi:10.1016/S0735-1097(02)01813-2.
4. Hinton, J.; Gabara, L.; Curzen, N. Is the true clinical value of high sensitivity troponins as a biomarker of risk? The concept that detection of high-sensitivity troponin "never means nothing." *Expert Rev. Cardiovasc. Ther.* 2020.
5. Gommans, D.H.F.; Cramer, G.E.; Fouraux, M.A.; Bakker, J.; Michels, M.; Dieker, H.J.; Timmermans, J.; Marcelis, C.L.M.; Verheugt, F.W.A.; de Boer, M.J.; et al. Prediction of Extensive Myocardial Fibrosis in Nonhigh Risk Patients With Hypertrophic Cardiomyopathy. *Am. J. Cardiol.* **2018**, doi:10.1016/j.amjcard.2018.04.020.
6. Hasegawa, K.; Fujiwara, H.; Doyama, K.; Miyamae, M.; Fujiwara, T.; Suga, S.; Mukoyama, M.; Nakao, K.; Imura, H.; Sasayama, S. Ventricular expression of brain natriuretic peptide in hypertrophic cardiomyopathy. *Circulation* **1993**, doi:10.1161/01.CIR.88.2.372.
7. Coats, C.J.; Gallagher, M.J.; Foley, M.; O'Mahony, C.; Critoph, C.; Gimeno, J.; Dawnay, A.; McKenna, W.J.; Elliott, P.M. Relation between serum N-terminal pro-brain natriuretic peptide and prognosis in patients with hypertrophic cardiomyopathy. *Eur. Heart J.* **2013**, doi:10.1093/eurheartj/eht070.
8. Geske, J.B.; McKie, P.M.; Ommen, S.R.; Sorajja, P. B-type natriuretic peptide and survival in hypertrophic cardiomyopathy. *J. Am. Coll. Cardiol.* **2013**, doi:10.1016/j.jacc.2013.04.004.
9. Stadiotti, I.; Pompilio, G.; Maione, A.S.; Pilato, C.A.; D'Alessandra, Y.; Sommariva, E. Arrhythmogenic cardiomyopathy: what blood can reveal? *Hear. Rhythm* 2019.
10. Kubo, T.; Ochi, Y.; Baba, Y.; Sugiura, K.; Takahashi, A.; Hirota, T.; Yamanaka, S.; Yamasaki, N.; Doi, Y.L.; Kitaoka, H. Elevation of high-sensitivity cardiac troponin T and left ventricular remodelling in hypertrophic cardiomyopathy. *ESC Hear. Fail.* **2020**, doi:10.1002/ehf2.12852.
11. Ponikowski, P.; Voors, A.A.; Anker, S.D.; Bueno, H.; Cleland, J.G.F.; Coats, A.J.S.; Falk, V.; González-Juanatey, J.R.; Harjola, V.-P.; Jankowska, E.A.; et al. 2016 ESC Guidelines for the diagnosis and treatment of acute and chronic heart failure: The Task Force for the diagnosis and treatment of acute and chronic heart failure of the European Society of Cardiology (ESC)Developed with the special contribution o. *Eur. Heart J.* **2016**, *37*, 2129–2200, doi:10.1093/eurheartj/ehw128.
12. Cheng, H.; Lu, M.; Hou, C.; Chen, X.; Wang, J.; Yin, G.; Chu, J.; Zhang, S.; Prasad, S.K.; Pu, J.; et al. Relation between N-terminal pro-brain natriuretic peptide and cardiac remodeling and function assessed by cardiovascular magnetic resonance imaging in patients with arrhythmogenic right ventricular cardiomyopathy. *Am. J. Cardiol.* **2015**, doi:10.1016/j.amjcard.2014.10.040.
13. Matsuo, K.; Nishikimi, T.; Yutani, C.; Kurita, T.; Shimizu, W.; Taguchi, A.; Suyama, K.; Aihara, N.; Kamakura, S.; Kangawa, K.; et al. Diagnostic value of plasma levels of brain natriuretic peptide in arrhythmogenic right ventricular dysplasia. *Circulation* **1998**, doi:10.1161/01.CIR.98.22.2433.
14. Gawor, M.; Śpiewak, M.; Janas, J.; Kożuch, K.; Wróbel, A.; Mazurkiewicz, Ł.; Baranowski, R.; Marczak, M.; Grzybowski, J. The usefulness of sST2 and galectin-3 as novel biomarkers for better risk stratification in hypertrophic cardiomyopathy. *Kardiol. Pol.* **2017**, doi:10.5603/KP.a2017.0118.
15. Yakar Tülüce, S.; Tülüce, K.; Çil, Z.; Volkan Emren, S.; İlke Akyıldız, Z.; Ergene, O. Galectin-3 levels in patients with hypertrophic cardiomyopathy and its relationship with left ventricular mass index and function. *Anatol. J. Cardiol.* **2016**, doi:10.5152/AnatolJCardiol.2015.6191.
16. Hu, D.J.; Xu, J.; Du, W.; Zhang, J.X.; Zhong, M.; Zhou, Y.N. Cardiac magnetic resonance and galectin-3 level as predictors of prognostic outcomes for non-ischemic cardiomyopathy patients. *Int. J. Cardiovasc. Imaging* **2016**, doi:10.1007/s10554-016-0958-1.
17. Vergaro, G.; Franco, A. Del; Giannoni, A.; Prontera, C.; Ripoli, A.; Barison, A.; Masci, P.G.; Aquaro, G.D.; Solal, A.C.; Padeletti, L.; et al. Galectin-3 and myocardial fibrosis in nonischemic dilated cardiomyopathy. *Int. J. Cardiol.* **2015**, doi:10.1016/j.ijcard.2015.02.008.
18. Oz, F.; Onur, I.; Elitok, A.; Ademoglu, E.; Altun, I.; Bilge, A.K.; Adalet, K. Galectin-3 correlates with arrhythmogenic right ventricular cardiomyopathy and predicts the

risk of ventricular arrhythmias in patients with implantable defibrillators. *Acta Cardiol.* **2017**, doi:10.1080/00015385.2017.1335371.
19. Montoro-García, S.; Hernández-Romero, D.; Jover, E.; García-Honrubia, A.; Vilchez, J.A.; Casas, T.; Martínez, P.; Climent, V.; Caballero, L.; Valdés, M.; et al. Growth differentiation factor-15, a novel biomarker related with disease severity in patients with hypertrophic cardiomyopathy. *Eur. J. Intern. Med.* **2012**, doi:10.1016/j.ejim.2011.08.022.
20. Stojkovic, S.; Kaider, A.; Koller, L.; Brekalo, M.; Wojta, J.; Diedrich, A.; Demyanets, S.; Pezawas, T. GDF-15 is a better complimentary marker for risk stratification of arrhythmic death in non-ischaemic, dilated cardiomyopathy than soluble ST2. *J. Cell. Mol. Med.* **2018**, doi:10.1111/jcmm.13540.
21. Lok, S.I.; Winkens, B.; Goldschmeding, R.; Van Geffen, A.J.P.; Nous, F.M.A.; Van Kuik, J.; Van Der Weide, P.; Klöpping, C.; Kirkels, J.H.; Lahpor, J.R.; et al. Circulating growth differentiation factor-15 correlates with myocardial fibrosis in patients with non-ischaemic dilated cardiomyopathy and decreases rapidly after left ventricular assist device support. *Eur. J. Heart Fail.* **2012**, doi:10.1093/eurjhf/hfs120.
22. Akdis, D.; Chen, L.; Saguner, A..; Zhang, N.; Gawinecka, J.; Saleh, L.; Von Eckardstein, A.; Ren, J.; Matter, C.; Hu, Z.; et al. Novel plasma biomarkers in arrhythmogenic cardiomyopathy: the role of ST2 and GDF-15 in predicting biventricular involvement. *Eur. Heart J.* **2020**, doi:10.1093/ehjci/ehaa946.0728.
23. Lichtenauer, M.; Jirak, P.; Wernly, B.; Paar, V.; Rohm, I.; Jung, C.; Schernthaner, C.; Kraus, J.; Motloch, L.J.; Yilmaz, A.; et al. A comparative analysis of novel cardiovascular biomarkers in patients with chronic heart failure. *Eur. J. Intern. Med.* **2017**, doi:10.1016/j.ejim.2017.05.027.
24. Binas, D.; Daniel, H.; Richter, A.; Ruppert, V.; Schlüter, K.-D.; Schieffer, B.; Pankuweit, S. The prognostic value of sST2 and galectin-3 considering different aetiologies in non-ischaemic heart failure. *Open Hear.* **2018**, doi:10.1136/openhrt-2017-000750.
25. Broch, K.; Leren, I.S.; Saberniak, J.; Ueland, T.; Edvardsen, T.; Gullestad, L.; Haugaa, K.H. Soluble ST2 is associated with disease severity in arrhythmogenic right ventricular cardiomyopathy. *Biomarkers* **2017**, doi:10.1080/1354750X.2016.1278266.
26. Roncarati, R.; Viviani Anselmi, C.; Losi, M.A.; Papa, L.; Cavarretta, E.; Da Costa Martins, P.; Contaldi, C.; Saccani Jotti, G.; Franzone, A.; Galastri, L.; et al. Circulating miR-29a, among other up-regulated microRNAs, is the only biomarker for both hypertrophy and fibrosis in patients with hypertrophic cardiomyopathy. *J. Am. Coll. Cardiol.* **2014**, doi:10.1016/j.jacc.2013.09.041.
27. Marinas, M.B.; Celeghin, R.; Cason, M.; Bariani, R.; Frigo, A.C.; Jager, J.; Syrris, P.; Elliott, P.M.; Bauce, B.; Thiene, G.; et al. A microRNA expression profile as non-invasive biomarker in a large arrhythmogenic cardiomyopathy cohort. *Int. J. Mol. Sci.* **2020**, doi:10.3390/ijms21041536.
28. Vilela, E.M.; Bettencourt-Silva, R.; da Costa, J.T.; Barbosa, A.R.; Silva, M.P.; Teixeira, M.; Primo, J.; Ribeiro, V.G.; Nunes, J.P.L. Anti-cardiac troponin antibodies in clinical human disease: A systematic review. *Ann. Transl. Med.* 2017.
29. Pinto, Y.M.; Elliott, P.M.; Arbustini, E.; Adler, Y.; Anastasakis, A.; Böhm, M.; Duboc, D.; Gimeno, J.; De Groote, P.; Imazio, M.; et al. Proposal for a revised definition of dilated cardiomyopathy, hypokinetic non-dilated cardiomyopathy, and its implications for clinical practice: A position statement of the ESC working group on myocardial and pericardial diseases. *Eur. Heart J.* **2016**, doi:10.1093/eurheartj/ehv727.
30. Caforio, A.L.P.; Mahon, N.G.; Baig, M.K.; Tona, F.; Murphy, R.T.; Elliott, P.M.; McKenna, W.J. Prospective familial assessment in dilated cardiomyopathy: Cardiac autoantibodies predict disease development in asymptomatic relatives. *Circulation* **2007**, doi:10.1161/CIRCULATIONAHA.106.641472.
31. Chatterjee, D.; Pieroni, M.; Fatah, M.; Charpentier, F.; Cunningham, K.S.; Spears, D.A.; Chatterjee, D.; Suna, G.; Martjin Bos, J.; Ackerman, M.J.; et al. An autoantibody profile detects Brugada syndrome and identifies abnormally expressed myocardial proteins. *Eur. Heart J.* **2020**, doi:10.1093/eurheartj/ehaa383.
32. Stiles, M.K.; Wilde, A.A.M.; Abrams, D.J.; Ackerman, M.J.; Albert, C.M.; Behr, E.R.; Chugh, S.S.; Cornel, M.C.; Gardner, K.; Ingles, J.; et al. 2020 APHRS/HRS expert consensus statement on the investigation of decedents with sudden unexplained death and patients with sudden cardiac arrest, and of their families. *Hear. Rhythm* **2021**, doi:10.1016/j.hrthm.2020.10.010.
33. Fu, M.L.X.; Hoebeke, J.; Matsui, S.; Matoba, M.; Magnusson, Y.; Hedner, T.; Herlitz, H.; Hjalmarson, Å. Autoantibodies against Cardiac G-protein-coupled receptors define different populations with cardiomyopathies but not with hypertension. *Clin. Immunol. Immunopathol.* **1994**, doi:10.1006/clin.1994.1101.
34. Peukert, S.; Fu, M.L.X.; Eftekhari, P.; Poepping, I.; Voss, A.; Thalhammer, C.; Hempel, A.; Menz, M.; Dietz, R.; Osterziel, K.J. The frequency of occurrence of anti-cardiac

receptor autoantibodies and their correlation with clinical manifestation in patients with hypertrophic cardiomyopathy. *Autoimmunity* **1999**, doi:10.3109/08916939908994749.

35. Duan, X.; Liu, R.; Luo, X.L.; Gao, X.J.; Hu, F.H.; Guo, C.; Wang, J.; Hu, X.Y.; Chun, Y.S.; Yuan, J.S.; et al. The relationship between β1-adrenergic and M2-muscarinic receptor autoantibodies and hypertrophic cardiomyopathy. *Exp. Physiol.* **2020**, doi:10.1113/EP088263.
36. Chatterjee, D.; Fatah, M.; Akdis, D.; Spears, D.A.; Koopmann, T.T.; Mittal, K.; Rafiq, M.A.; Cattanach, B.M.; Zhao, Q.; Healey, J.S.; et al. An autoantibody identifies arrhythmogenic right ventricular cardiomyopathy and participates in its pathogenesis. *Eur. Heart J.* **2018**, doi:10.1093/eurheartj/ehy567.
